# Effect of Mowing on Wheat Growth at Seeding Stage

**DOI:** 10.3390/ijms242015353

**Published:** 2023-10-19

**Authors:** Song Li, Shaoyu Wang, Wenjie Ye, Yaxin Yao, Fengli Sun, Chao Zhang, Shudong Liu, Yajun Xi

**Affiliations:** College of Agronomy, Northwest A&F University, Yangling 712100, China; 15619357797@163.com (S.L.); 18715152017@163.com (S.W.); ye453326614@163.com (W.Y.); yaoyaxin_sxau@163.com (Y.Y.); sunof1981@126.com (F.S.); ahzc2009@163.com (C.Z.); liushd325@163.com (S.L.)

**Keywords:** mowing, growth patten, hormone, transcriptome, gene expression pattern

## Abstract

Winter wheat is used as forage at the tillering stage in many countries; however, the regrowth pattern of wheat after mowing remains unclear. In this study, the growth patterns of wheat were revealed through cytological and physiological assessments as well as transcriptome sequencing. The results of agronomic traits and paraffin sections showed that the shoot growth rate increased, but root growth was inhibited after mowing. The submicroscopic structure revealed a decrease in heterochromatin in the tillering node cell and a change in mitochondrial shape in the tillering node and secondary root. Analysis of the transcriptome showed the number of differentially expressed genes (DEGs) involved in biological processes, cellular components, and molecular functions; 2492 upregulated DEGs and 1534 downregulated DEGs were identified. The results of the experimental study showed that mowing induced expression of DEGs in the phenylpropanoid biosynthesis pathway and increased the activity of PAL and 4CL. The upregulated DEGs in the starch and sucrose metabolism pathways and related enzyme activity alterations indicated that the sugar degradation rate increased. The DEGs in the nitrogen metabolism pathway biosynthesis of the amino acids, phenylpropanoid biosynthesis metabolism, and in the TCA pathway also changed after mowing. Hormone content and related gene expression was also altered in the tillering and secondary roots after mowing. When jasmonic acid and ethylene were used to treat the wheat after mowing, the regeneration rate increased, whereas abscisic acid inhibited regrowth. This study revealed the wheat growth patterns after mowing, which could lead to a better understanding of the development of dual-purpose wheat.

## 1. Introduction

Dual-purpose wheat refers to wheat being mowed at the tillering stage to use the shoot biomass as livestock forage, with the grain harvested in summer. Ideally, dual-purpose wheat provides greater forage for livestock in winter without decreasing grain yield in summer. This wheat management system is found across different regions and countries, such as Australia, the Americas, and Europe [1,2,3], but it is rarely reported in China [4]. With improvements in living standards, the demand for livestock products is increasing. Due to the distinctive seasons, the production of forage in winter cannot meet the needs of livestock in northern China. Shortage of forage restricts the development of livestock farming in northern China [5,6]. Therefore, finding reliable and high-quality forage is the key to addressing this problem. Northern China is characterized by large areas under wheat cultivation, which could provide large amounts of shoot biomass at the tillering stage, and the coarse fiber content of the wheat is low, allowing for easy digestion of high-quality forage by grazing livestock. Similarly, some studies have verified that wheat has a strong regeneration ability at the tillering stage, and the yield of winter wheat does not decrease in summer [7,8]. However, the focus of most studies were the effects of mowing frequency and stubble height on wheat regeneration, and many researches have also focused on the de novo regeneration of plants; the regeneration pattern of wheat after mowing remains unclear [9].

In plants, growth patterns and organ function are continually adjusted after damage, such as herbivory, and this regrowth mechanism is vital for plant survival [10,11]. The physiological feedback system of plants coordinates numerous metabolic processes during regrowth and maintains a state of relative balance [10,12]. The regrowth of wheat after mowing involves plant photosynthetic organ repair, namely, leaf growth and expansion. During this process, auxins (IAA) affects leaf formation and development [13,14], whereas cytokinins (CTK) maintain the quantity of stem cells in the shoot apical meristem (SAM) and play a critical role in plant leaf development [15,16]. Gibberellin (GA) promotes cell expansion during leaf development [17,18]. In plants such as potato (*Solanum tuberosum*), tomato (*Solanum Lycopersicum*), and tobacco (*Nicotiana Tabacum*), abscisic acid (ABA) can induce the expression of the injury response gene PINII [19], which indicates that ABA may also be involved in injury regrowth in plants. In cotton, the ABA treatment of somatic embryos induces the expression of *LEC1*, *FUS3*, and *WUSCH1*, increasing the number of cotyledon embryos [20]. Recent studies have shown that jasmonic acid (JA) also plays an important role in plant regeneration [21,22]. When the plant is injured, the transcriptional inhibition of gene *LOX2* is relieved, and the JA content increases in response to the injury [23]. JA stimulates auxin synthesis in plants via long-distance signal transduction during plant regrowth process [24]. Ethylene (ETH) is an essential hormone that regulates plant growth and development and responds to injury [25]. It has been verified that *ACS2,6,7,8* responded rapidly to injury in the leaves of *Arabidopsis thaliana* [26,27].

A previous study has proved that the transcriptome is analyzed after mowing to reveal the regrowth mechanism of the plants. There are several changes in the expression of many genes in grasses, with the majority of these changes concentrated on cellular components, molecular functions, and biological processes [28]. One of the most important pathways in plant recovery after mowing is phenylpropanoid metabolism, which is the primary pathway for the synthesis of phenolic compounds [29]. Some studies have shown that injury triggers the synthesis of phenylalanine and phenolic compounds [29,30]. Sugar metabolism provides an energy and carbon framework for plant regeneration, such as amino acid synthesis [31], and an increase in reducing sugar content causes an increase in amino acid content [32,33]. Additionally, nitrogen metabolism provides a nitrogen framework for amino acid synthesis following wounding [34]. The tricarboxylic acid (TCA) cycle is essential for both the synthesis of biomolecules and generation of ATP, which also provides energy and C/N fragments for plant regrowth [35]. In plants, the speed of the TCA increases after injury [36]; plant metabolism is adjusted to promote regeneration.

The application of dual-purpose wheat not only relieves the forage shortage in winter but also increases the effectiveness of land use and provides environmental protection. Plant regrowth is the key factor for dual-purpose wheat used as forage in the tillering stage [37]. Dual-purpose wheat guarantees high wheat yield in summer and provides fresh forage during winter. However, most studies are focus on the effects of cultivation conditions and soil nutrients on the yield and quality of forage and grain, and few studies have reported regeneration mechanisms after dual-purpose wheat mowing [1,38]. Therefore, it would be advantageous to understand the growth patterns of dual-purpose wheat after mowing, to aid in wheat crop management in China. In this study, the “XiNong136” was used to analyze the growth pattern after mowing by analyzing the changes in cytology, transcriptome, and related physiology. The results provide a theoretical foundation for the utilization of dual-purpose wheat in China.

## 2. Results

### 2.1. Shoot and Root Phenotype Analysis

**Figure 1:**
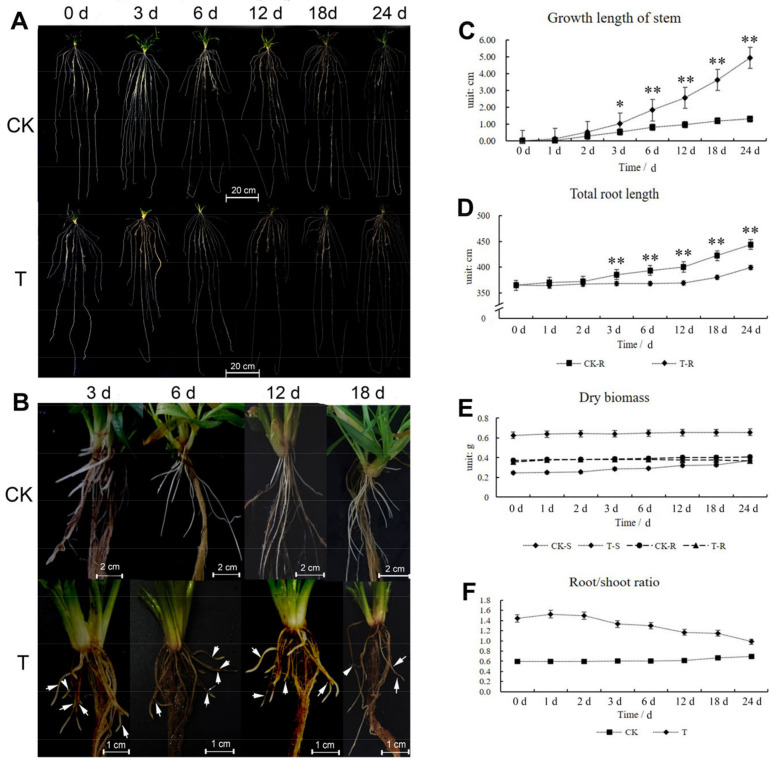
The agronomics traits of shoot and root of wheat after mowing. (**A**) The phenotype of shoot and root after mowing, bar = 20 cm. (**B**) The phenotype of secondary root after mowing, bar = 2 cm. White arrows indicated the shrink of secondary root. (**C**) The growth length of stem. (**D**) The total root length of T and CK. (**E**) Dry biomass of stem and root. (**F**) Root/shoot ratio. CK: control group. T: treatment group. CK-S: wheat stem of control group. T-S: wheat stem of treatment group. CK-R: secondary root of control group. T-R: wheat root of treatment group. The error bars indicated standard deviations. “*” and “**” represent *p* < 0.05 or *p* < 0.01 (Student’s *t*-test).

During winter, wheat root growth was found to accelerate, whereas the leaves almost stopped growing. However, mowing the shoots resulted in a different developmental pattern and caused the root diameter of some secondary roots to be smaller than that of the control group (CK) (Figure 1A,B); after mowing, the growth rate of the shoots increased, but root growth was restrained. Investigation of shoot agronomic traits showed that the shoot growth length of the CK was 1.3 cm, which was significantly lower than the corresponding measure in the treatment group (4.94 cm) (Figure 1C). However, the total root growth length of CK was 79.5 cm, which was significantly longer than the 34.73 cm in the treatment group (T) within 24 d (day) (Figure 1D). Shoot and root dry biomass weights also changed. The shoot dry biomass of CK increased by 0.030 g, which was significantly lower than the 0.128 g in T after mowing, but the root dry biomass of CK increased by 0.035 g and that of T decreased by 0.006 g (Figure 1E). The root/shoot ratio in CK and T exhibited a progressive increase in CK and a gradual decrease in T (Figure 1F).

The length of the longest root and the number of root primordia and secondary roots in CK and T were also measured. These results also showed that mowing restrained root growth (Figure S1A–C). Within 24 d, the longest root in CK increased by nearly 17 cm, whereas in T increase by 5.67 cm (Figure S1A). Approximately 6 secondary roots increased in CK, whereas only 2.4 secondary roots increased in T, root primordia in T decreased, while it increased in CK (Figure S1B). After 2 d of mowing, the surface area of the roots in T was considerably lower than that of CK (Figure S1C). The average root length decreased in T and CK, and after 18 d, the average root length of CK was significantly higher than that of T, whereas the average root length of the new secondary roots decreased (Figure S1D). Shoot and root phenotype analyses showed that mowing wheat inhibited root growth but accelerated shoot growth.

### 2.2. Paraffin Section Analysis of Tillering Node after Mowing

**Figure 2:**
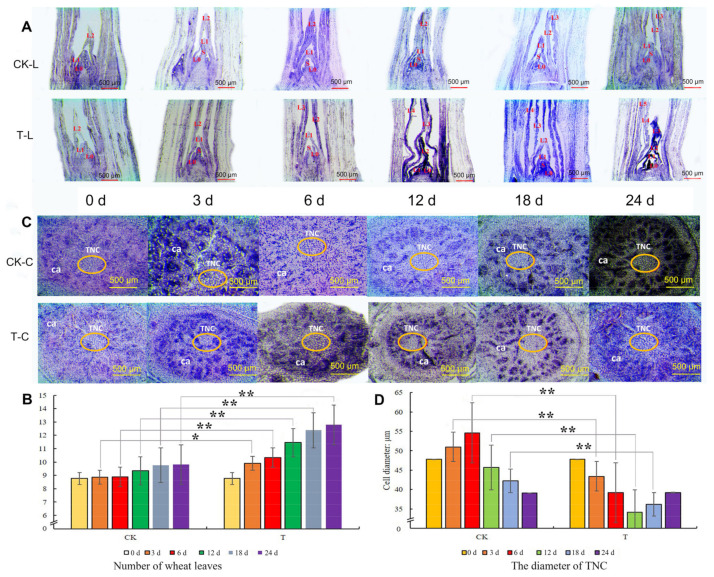
Paraffin section of wheat TN. (**A**) The length cutting of tillering node, bar = 500 μm. (**B**) Number of wheat leaves of T and CK. (**C**) The cross cutting of tillering node, bar = 500 μm. (**D**) The diameter of tillering node cells. CK-L: length cutting of control group. T-L: length cutting treatment group. CK-C: crosscut of control group. T-C: crosscut cutting treatment group. L0-L5: the new leaves in wheat main stem. S: shoot apical meristem. ca: cambium. TNC: cell of tillering node. The error bars indicate standard deviations. “*” and “**” represent *p* < 0.05 or *p* < 0.01 between CK and T (Student’s *t*-test).

Paraffin sections were analyzed to ascertain the wheat leaf growth patterns after mowing. As shown in Figure 2, new leaves appeared more rapidly in the T than in CK. At 3 d and 6 d after mowing, T plants had three new leaves, whereas CK plants had only two leaves (Figure 2A). Twelve days after mowing, T formed one new leaf, resulting in T having four leaves, whereas CK still had two leaves (Figure 2A). On day 24, T had five new leaves, whereas CK had three (Figure 2A). The total number of wheat leaves was then determined. Three days after mowing, the number of leaves in T was significantly higher than that in CK (Figure 2B). These results demonstrated that the rate of leaf formation in T was faster than in CK.

The tillering node (TN) structure remained unchanged even when the pace of fresh leaf development increased. As shown in Figure 2C, the cambium (ca) in the T and CK was not significantly changed. However, the cell volume of the TN (TNC) changed after mowing. At 3 and 6 d, the cell diameter increased under normal growth conditions, whereas it decreased in T, and the cell diameter of CK was significantly larger than that of T (Figure 2C,D). At day 12, the cell diameters of TNC in CK and T decreased, but CK was still significantly larger than that of T (Figure 2C,D). After 18 d of mowing, the cell diameter of TNC in T increased, whereas that in CK decreased (Figure 2D). The cell diameter of TNC changed in the T and CK groups, and mowing might accelerate cell division.

### 2.3. Paraffin Section Analysis of Root after Mowing

**Figure 3:**
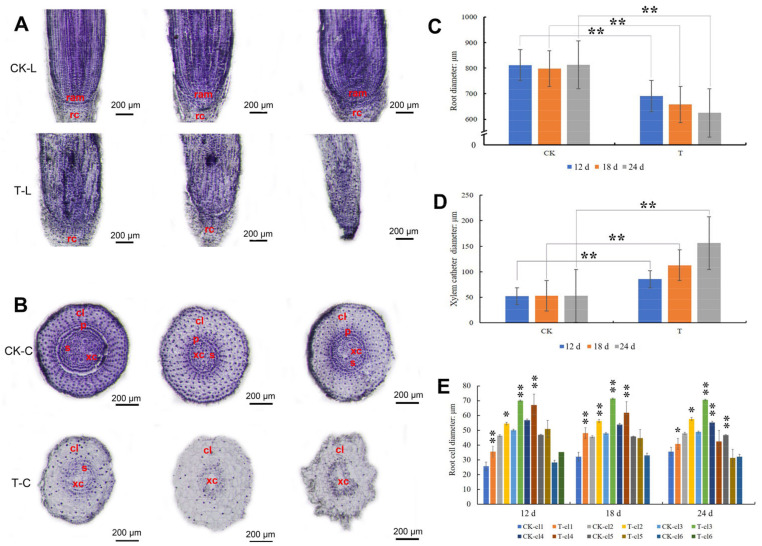
Paraffin section of wheat SRT. (**A**) The length cutting of secondary root tip, bar = 200 μm. (**B**) The cross cut-ting of secondary root tip, bar = 200 μm. (**C**) The diameter of secondary root of CK and T. (**D**) The xylem catheter di-ameter of CK and T. (**E**) The cortex layer cell diameter. CK-L: length cutting of secondary root of control group. T-L: length cutting secondary root of treatment group. CK-C: crosscut of secondary root of control group. T-C: crosscut of secondary root of treatment group. T-cl: the cortex layer of treatment group. CK-cl: the cortex layer of control group, cl1-cl6: the cortex layer location. ram: root apical meristem, rc: root cap, cl: cortex layer, s: stele, xc: xylem catheter, p: pericycle. The error bars indicated standard deviations. “*” and “**” represent *p* < 0.05 or *p* < 0.01 between CK and T (Student’s *t*-test).

Root paraffin sections were analyzed to determine the root development patterns after mowing. The results showed that the structure of the primary root did not change after mowing, although the primary root length in T was shorter than that in CK (Figure S2A–D). However, the tip of the secondary root (SRT) changed after mowing. When comparing the root structures of T and CK at 0, 3, and 6 d, there were no differences (Figure S3); however, after 12 d, some SRT had shrunk, resulting in the root diameter of T being significantly smaller than that of CK, and an unorganized interior structure (Figure 3A–C). At 24 d, the root pericycle, root stele, root apical meristem, and root cap of the secondary root disappeared, and the root form was abnormal (Figure 3A,B). After mowing, the xylem catheter in T progressively increased and was significantly larger than that in CK after 6 d (Figure 3D). At 18 and 24 d, T had fewer cortical layers than before, and there were five cortical layers in T as opposed to six in CK (Figure 3E). Although there were fewer cortical layers in T, the diameter of some of the cortical layer cells had expanded considerably after mowing (Figure 3E).

### 2.4. Sub-Microstructure of Wheat Tillering Node and Secondary Root after Mowing

**Figure 4:**
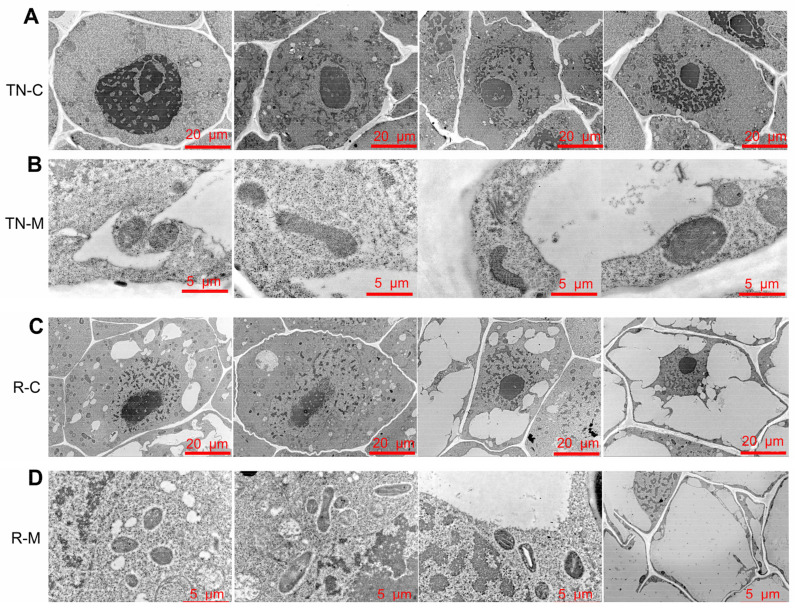
Sub-microstructure of wheat TN and SRT. (**A**) Sub-microstructure of wheat tillering node cells, bar = 20 μm. (**B**) Sub-microstructure of mitochondria in tillering node, bar = 5 μm. (**C**) Sub-microstructure of secondary root tip, bar = 20 μm. (**D**) Sub-microstructure of mitochondria in secondary root tip, bar = 5 μm. TN-C: sub-microstructure of tillering node cell. TN-M: sub-microstructure of mitochondria in tillering node. R-C: sub-microstructure of SRT cell. R-M: sub-microstructure of mitochondria in secondary root tip.

To analyze internal alterations in the cells, the subcellular structures of the TNC and secondary roots were observed. The results showed that the chromatin filaments in TN cells were packed and folded tightly and presented as agglutinated heterochromatin at 0 d (Figure 4A). After mowing, the heterochromatinization decreased at 3 and 6 d, while increasing at 12 d, but the heterochromatinization remained lower than that at 0 d (Figure 4A). These results indicate that the transcription of genes in TN might increase. In addition, wheat nucleoli showed a spongy appearance after mowing (Figure 4A). Mitochondria were observed, and the results showed that the shape of the mitochondria changed from oval to rod-like after mowing and the volume increased. However, after 12 d, the mitochondria changed to an oval shape (Figure 4B). Changes in the mitochondria and heterochromatin indicated that cell activity in the TN increased from 3 d to 12 d after mowing.

The sub-microstructure of root cells was similarly affected by mowing. As shown in Figure 4C, under normal growth conditions, chromosome heterochromatin was low, suggesting a high level of SRT activity in the cells (Figure 4C). At 3 and 6 d after mowing, heterochromatin levels were reduced. The shape of mitochondria in roots showed the same alteration as TN at 3 d, but at 6 d, the mitochondria of SRT assumed an oval shape and the volume was lower than at 0 d (Figure 4D). The majority of the intracellular organelles in the root cells vanished after 12 d. These results suggested that mowing increased cell activity at an early stage and results in the death of secondary root cells at a later stage.

### 2.5. Transcriptome Analysis of the DEGs in TN

**Figure 5:**
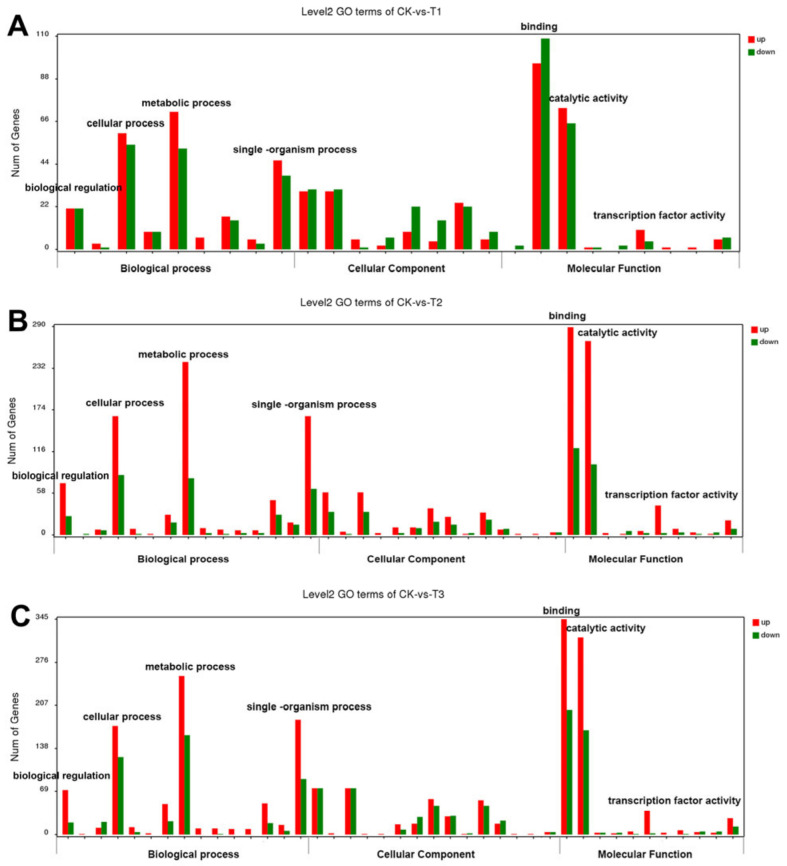
Gene ontology analysis of upregulated and downregulated DGEs. (**A**) CK: T1 (0 h:2 h), (**B**) CK: T2 (0 h:24 h), (**C**) CK: T3 (0 h:72 h).

The transcriptome of TN was examined within 3 d of mowing for the results of the sub-microstructure and agronomic traits changed at the 3 d after mowing. Additionally, TN samples at 2 h after mowing were selected to analyze the effects of mowing on wheat at an early stage. After filtering the low-quality data, approximately 18.6 Gb of sequence data for each sample were obtained. A total of 4476 DEGs (differentially expressed genes) were identified between CK and T (T1-T3), including 2942 upregulated and 1534 downregulated genes. As shown in Figure 5, according to the gene ontology (GO) annotation, the DEGs can be divided into three groups: biological processes, cellular components, and molecular functions (Figure 5). There were 397, 1101, and 1444 DEGs that were upregulated and 388, 477, and 669 DEGs downregulated in CK at T1 (2 h), CK: T2 (24 h), and CK: T3 (72 h), respectively (Figure 5A–C). The majority of DEGs focused on biological processes and molecular functions, and they were numerous at 24 and 72 h compared to 2 h after mowing (Figure 5A–C). In terms of biological processes, DEGs were concentrated in biological regulation, cellular processes, metabolic processes, and signal–organism processes, and most genes were upregulated (Figure 5A–C). In terms of molecular functions, the DEGs were concentrated in binding, catalytic activity, and transcription factor activity. Most binding-related genes were downregulated at 2 h after mowing, whereas at 24 and 72 h, most DEGs were upregulated. The number of upregulated DEGs in molecular function also increased at 24 and 72 h after mowing.

### 2.6. KEGG Pathway Analysis of Phenylpropanoid Biosynthesis

**Figure 6:**
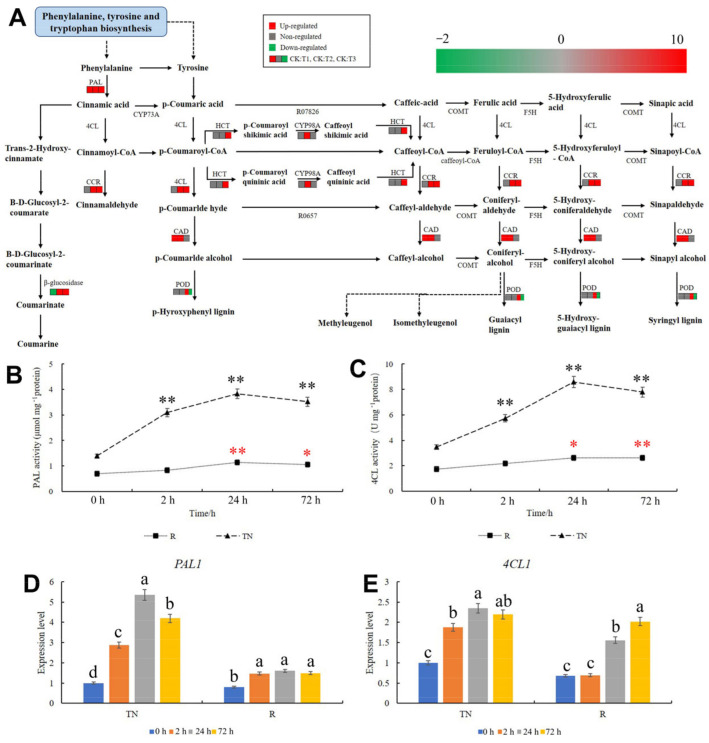
Changes in the phenylpropanoid biosynthesis pathway in TN. (**A**) KEGG pathway analysis of phenylpropanoid biosynthesis. Red indicates upregulation; green indicates downregulation; and gray indicates no significant difference. (**B**) PAL activity of TN and SR, (**C**) 4CL activity of TN and SR, (**D**) The expression pattern of *PAL1* in TN and SR after mowing. (**E**) The expression pattern of *4CL1* in TN and SR after mowing. TN: tillering node. SR: secondary root. The error bars indicated standard deviations. Black and red “*/“**” represent “*p* < 0.05” or “*p* < 0.01” in tillering node or secondary root, respectively. Difference in lowercase represents the significant difference of gene expression level.

PAL is the first key enzyme in polyphenol synthesis, protecting wheat by preventing the invasion of pathogenic agents. The genes in the PAL pathway were upregulated after mowing (Figure 6A, Table S1), according to the KEGG pathway analysis of phenylpropanoid biosynthesis, which resulted in an accumulation of cinnamic acid in the TN and more substrates for the downstream processes that are catalyzed by 4CL and CYP73A. DEGs in the cinnamoyl–CoA reductase (CCR), CAD, and POD metabolic pathways were also analyzed after mowing in the phenylpropanoid biosynthesis pathway. The genes in CCR metabolism were upregulated at 24 and 72 h, whereas in the CAD metabolism pathway, the genes were upregulated at 2 and 24 h (Figure 6A, Table S1). The expression of genes involved in the POD metabolism pathway was unaffected at 2 and 24 h after mowing. However, after 72 h, four genes were downregulated and five genes were upregulated (Figure 6A, Table S1). PAL activity in the TN and secondary roots (SR) was detected, and the results showed that PAL activity increased (Figure 6B). The activity of 4CL also increased the TN and SR after mowing (Figure 6C). Moreover, the expression of *PAL1* in DEGs in the PAL metabolism pathway was measured, and the results showed that it was significantly upregulated in TN and SR after mowing (Figure 6D, Table S2), which indicated that the results of transcriptome sequencing were reliable. Although no DEGs were detected in the 4CL metabolism pathway after mowing according to the sequencing, *4CL1* was upregulated in TN and SR (Figure 6D, Table S2).

### 2.7. KEGG Pathway Analysis of Carbohydrate Metabolism

**Figure 7:**
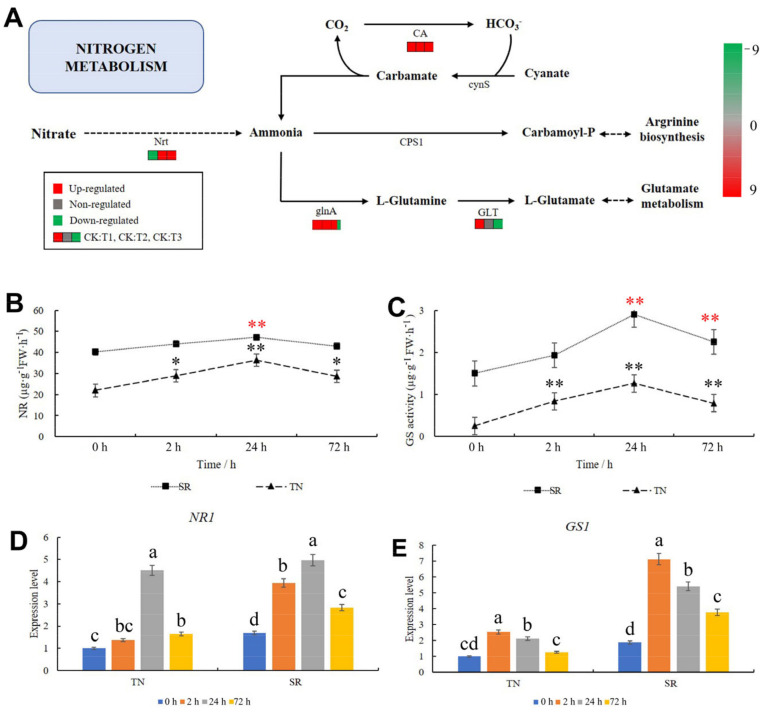
Changes in the starch and sucrose metabolism in TN. (**A**) KEGG pathway analysis of starch and sucrose metabolism. Red indicates upregulation; green indicates downregulation; and gray indicates no significant difference. (**B**) The activity of β-Bmybase. (**C**) The activity of β-glucosidase. (**D**) The expression pattern of *BAM1* in TN and SR after mowing. (**E**) The expression pattern of *BGL1* in TN and SR after mowing. TN: tillering node. SR: secondary root. The error bars indicated standard deviations. Black and red “*”/“**” represent “*p* < 0.05” or “*p* < 0.01” in TN and SR, respectively. Difference in lowercase represents the significant difference of gene expression level.

Starch and reducing sugar degradation provide energy for wheat re-growth after mowing. In this study, the carbohydrate metabolism in the TN after mowing was analyzed. The results showed that in the starch and sucrose pathways, there were 68 DEGs in CK vs. T1, T2, and T3, with only two genes were downregulated at 2 h in the sucrose synthase (SUS) and β-glucosidase (bglX/B) metabolism pathways and 50 genes were upregulated at 24 and 72 h in other metabolic pathways (Figure 7A, Table S3). The genes in β-Bmybase, SUS, bglX/B, trehalose 6-phosphate synthase (otsA), and trehalose 6-phosphate phosphatase (otsB) metabolism pathways were upregulated at 24 and 72 h after mowing (Figure 7A), indicating that starch and glucose were hydrolyzed and provided energy and carbon fragments for shoot regeneration. The β-Bmybase and β-glucosidase activities were detected to reveal the starch and glucose hydrolyzation rate. The results showed that the β-Bmybase and β-glucosidase activities increased in the TN after mowing. The activities of these two enzymes in the SR significantly increased at 2 and 24 h but decreased at 72 h after mowing (Figure 7B,C). Later, the expression of *BAM1* and *BGL2* in DGEs in the sugar and sucrose metabolism pathways was detected with qRT-PCR, and the results indicated that these two genes were upregulated at various intervals in both TN and SR after mowing (Figure 7D,E, Table S2).

### 2.8. KEGG Pathway Analysis of Nitrogen Metabolism

**Figure 8:**
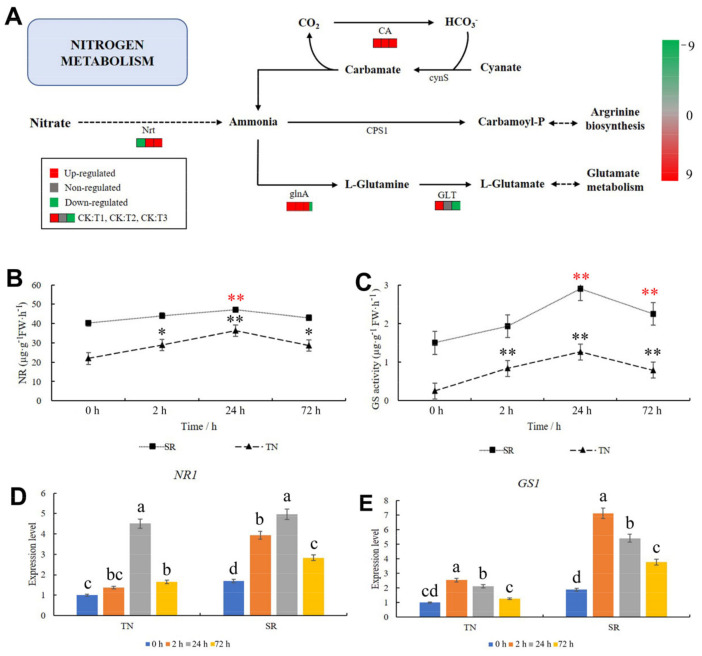
Changes in the nitrogen metabolism in TN. (**A**) KEGG pathway analysis of nitrogen metabolism. Red indicates upregulation; green indicates downregulation; and gray indicates no significant difference. (**B**) The activity of NR. (**C**) The activity of GS. (**D**) The expression pattern of *NR1* in TN and SR after mowing. (**E**) The expression pattern of *GS1* in TN and SR after mowing. TN: tillering node. SR: secondary root. The error bars indicated standard deviations. Black and red “*”/“**” represent “*p* < 0.05” or “*p* < 0.01” in TN and SR, respectively. Difference in lowercase represents the significant difference of gene expression level.

Nitrogen metabolism was analyzed to determine the capacity of wheat to convert nitrogen after mowing. As shown in Figure 8A, two genes in the nitrate transporter (Nrt) metabolism pathway were downregulated at 2 h and upregulated at 24 and 72 h (Figure 8A, Table S4). This result suggests that more ammonia would accumulate in the TN and act as a substrate for the downstream metabolic pathway. In the downstream pathways, two genes in the glutamine synthetase (glnA) metabolism pathway were upregulated at 2 and 24 h, whereas one gene was downregulated at 72 h (Figure 8A, Table S4). Six genes in the carbonic anhydrase (CA) metabolic pathway were upregulated after mowing (Figure 8A, Table S4). Then, the nitrate reductase (NR) and glutamine synthetase (GS) activities were measured, and the results showed that these two enzymes were significantly increased in the TN, whereas in the SR, they were significantly decreased at 24 and 24–72 h, respectively (Figure 8B,C). The expression patterns of *NR1* and *GS1* in the nitrogen metabolism pathway were determined through qRT-PCR. The results showed that the expression patterns of *NR1* and *GS1* in TN were consistent with those observed in the transcriptome. These two genes were upregulated after mowing (Figure 8D,E, Table S2). These results suggested that the nitrogen metabolism rate in wheat was enhanced after mowing.

### 2.9. KEGG Pathway Analysis of Amino Acid Metabolism

**Figure 9:**
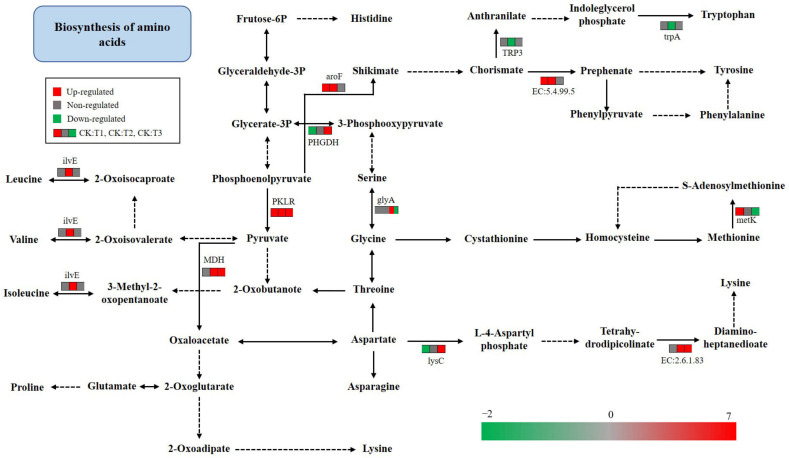
Changes in the biosynthesis of amino acids. Red indicates upregulation; green indicates downregulation; and gray indicates no significant difference.

Amino metabolism was analyzed according to the KEGG pathway of transcriptome sequencing. A total of 37 upregulated DEGs were implicated in the biosynthesis of amino acids, including 9 in the pyruvate kinase metabolism pathway (PKLR), 9 in aminotransferase (ilvE), 6 in aroF, 6 in malic dehydrogenase (MDH), and 7 in the L-diaminopimelate aminotransferase metabolism pathway (EC:2.6.1.83) (Figure 9, Table S5). The upregulated genes in the PKLR metabolism pathway of TN after mowing might cause the pyruvate content to increase in TN, which is the substrate involved in the synthesis of leucine, valine, and isoleucine. Three amino acids may accumulate in the TN after mowing, given that nine genes in the ivlE metabolism pathway were upregulated (Figure 9). Six genes in the phenylalanine metabolic pathway were upregulated, resulting in an increase in phenylalanine content after mowing. (Figure 9) The lysine content might have increased for seven genes of EC:2.6.1.83 were upregulated.

**Figure 10:**
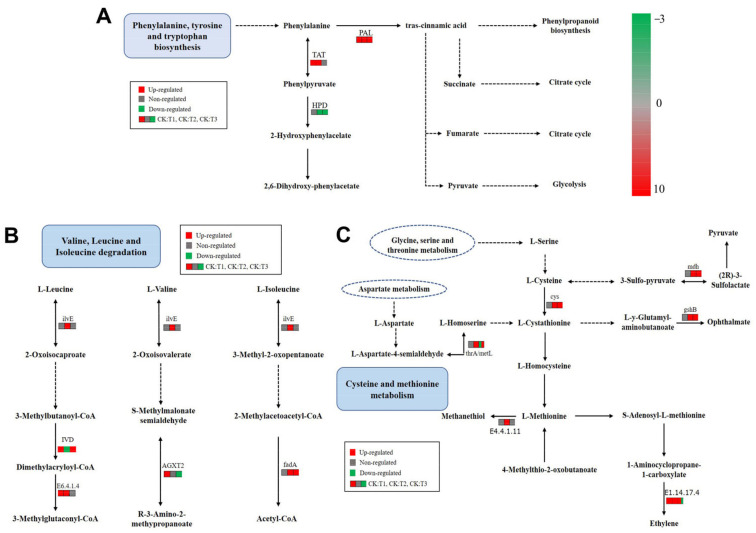
Changes in the different amino acid metabolisms. (**A**) KEGG pathway analysis of phenylalanine, tyrosine and tryptophan biosynthesis. (**B**) KEGG pathway analysis of valine, leucine and isoleucine. (**C**) KEGG pathway analysis of cysteine and methionine metabolism. Red indicates upregulation; green indicates downregulation; and gray indicates no significant difference.

Different amino acid metabolic pathways were analyzed to better understand amino acid metabolism after mowing. The results showed that one gene in the tyrosine aminotransferase (TAT) metabolism pathway was upregulated at 2 and 24 h after mowing, suggesting that the rate of tyrosine and phenylpyruvate metabolism increased (Figure 10A, Table S6). As shown in Figure 10B, mowing induced the genes expression in the branched-chain amino acid aminotransferase (ilvE), isovaleryl-CoA dehydrogenase (IVD), acetyl-CoA acyltransferase (fadA), and E6.4.1.4A metabolism pathways, which may increase the degradation of branched chain amino acids and promote the production of precursor substances for glucosinolate biosynthesis (Figure 10B). In the cysteine and methionine metabolic pathways, 13 genes were upregulated at different time points, indicating that the synthesis rates of L-cystathionine, L-aspartate, and pyruvate increased (Figure 10C, Table S6). The synthesis rate of ethylene might also have increased, as indicated by the fact that two genes were upregulated after mowing (Figure 10C, Table S6).

### 2.10. KEGG Pathway Analysis of Citrate Cycle (TCA Cycle)

**Figure 11:**
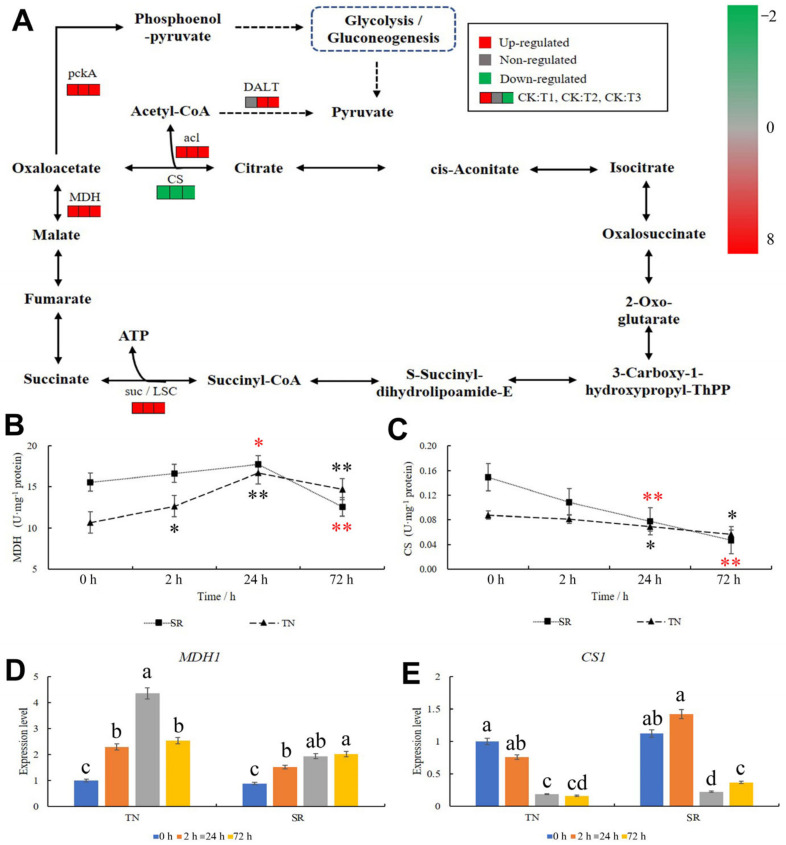
Changes in the TCA metabolism. (**A**) KEGG pathway analysis of TCA. Red indicates upregulation; green indicates downregulation; and gray indicates no significant difference. (**B**) The activity of MDH. (**C**) The activity of CS. (**D**) The expression pattern of *MDH1* in TN and SR. (**E**) The expression pattern of *CS1* in TN and SR after mowing. TN: tillering node. SR: secondary root. The error bars indicated standard deviations. Black and red “*”/“**” represent “*p* < 0.05” or “*p* < 0.01” in TN and SR, respectively. Difference in lowercase represents the significant difference of gene expression level.

The TCA cycle is a common oxidation pathway for sugars, fats, and proteins. In the TN, according to transcriptome sequencing, there were 11 genes in the TCA cycle after mowing, with 10 genes were upregulated and 1 was downregulated (Figure 11A, Table S7). The expression patterns of genes in the MDH and CS groups were the opposite: the concentration of oxaloacetate increased, as evidenced by the upregulation of three genes of MDH and the downregulation of one gene of CS (Figure 11A, Table S7). The content of acetyl-CoA and pyruvate may also increase for one gene in the acl metabolic pathway, and four genes were upregulated in the DALT metabolism pathway (Figure 11A, Table S7). After mowing, one gene was upregulated in the suc/LSC metabolic pathway, suggesting that the ATP production rate increased (Figure 11A). The activities of malate dehydrogenase (MDH) and citrate (Si)-synthase (CS) of the TN and SR were subsequently measured. The results showed that MDH activity increased in the TN after mowing, and this enzyme activity also significantly increased at 24 h in the SR but decreased at 72 h (Figure 11B). After mowing, CS activity in the TN and SR decreased (Figure 11C). Expression patterns of *MDH1* and *CS1* in the TCA metabolism pathway reflected the enzymatic activity (Figure 11D,E, Table S2).

### 2.11. Determination of Hormone Content in TN and SR

**Figure 12:**
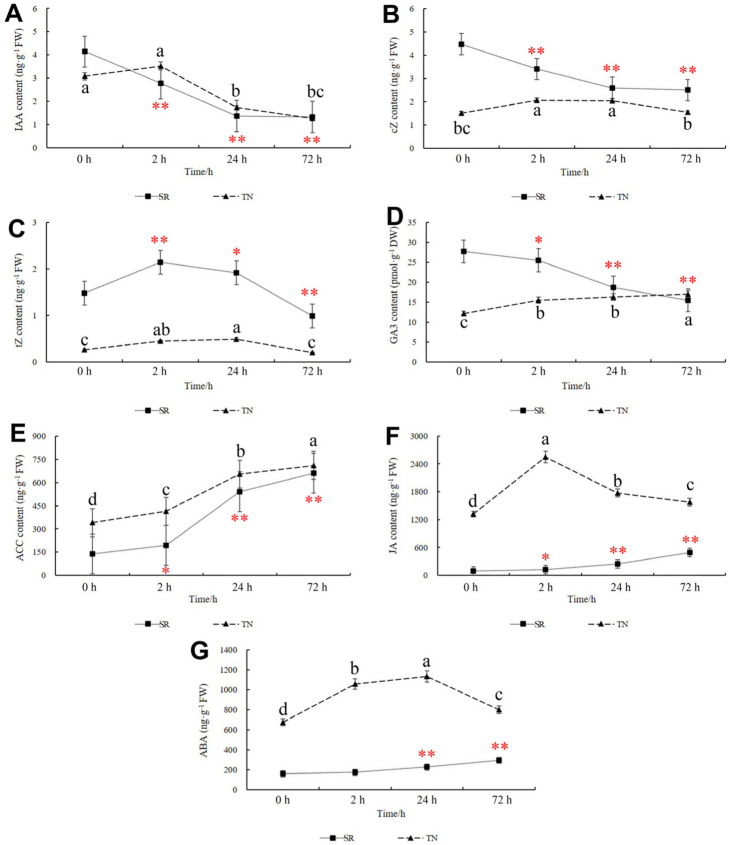
Mowing induced changes in hormone content. (**A**) IAA content in TN and SR. (**B**,**C**) cZ and tZ content in TN and SR. (**D**) GA3 content in TN and SR. (**E**) ACC content in TN and SR. (**F**) JA content in TN and SR. (**G**) ABA content in TN and SR. TN: tillering node. SR: secondary root. The error bars indicated standard deviations. Red “*”/“**” represent “*p* < 0.05” or “*p* < 0.01” in SR. Difference in lowercase represents the significant difference of hormone content in TN.

The contents of IAA, cZ, tZ, GA3, ACC, JA, and ABA were measured. The results showed that the IAA content in TN marginally increased at 2 h and significantly decreased at 24 and 72 h after mowing, whereas in SR, the IAA content significantly decreased after mowing (Figure 12A). Although the IAA content decreased in TN, the expression of genes associated with IAA synthesis and transport increased. In the SR, IAA-related genes showed different expression patterns (Figure S4A–C, Table S2). Trans-zeatin (tZ) content increased in TN and SR at 2 and 24 h after mowing but dropped at 72 h. The cis-zeatin (cZ) content increased in TN and decreased in SR (Figure 12B,C). The expression of *CKX*, *CISZOG*, and *IPT* changed in TN and SR after mowing (Figure S4D–F, Table S2). The GA3 content increased in the TN and decreased in the SR. Genes related to *GAMYB*, *GA20ox2*, and *GA30ox2* were upregulated in TN and SR at different time points after mowing (Figure S4G–I, Table S2).

The levels of ACC, JA, and ABA were also measured, and the results indicated that the levels of these three hormones were significantly increased in the TN and SR (Figure 12E–G). The variations in hormone-related gene expression variations correlated with changes in hormone levels (Figure S5, Table S2).

### 2.12. Regeneration and Gene Expression Pattern under ETH, MeJA, and ABA Treatment

**Figure 13:**
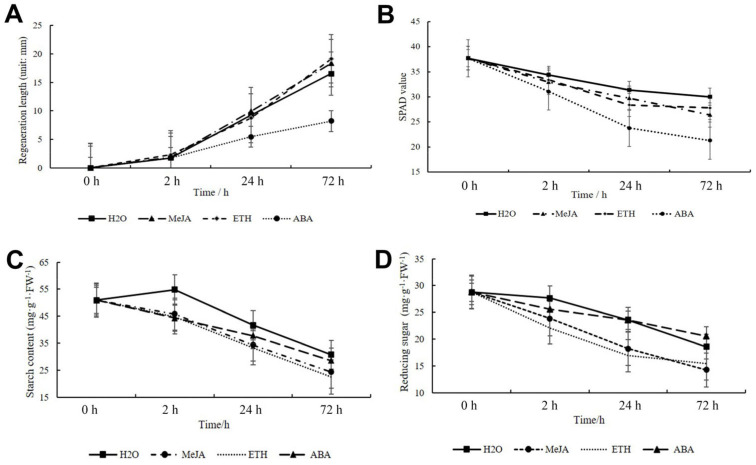
The wheat regeneration pattern after ABA, ETH, and JA treatment. (**A**) The shoot regeneration length under normal condition and different hormones treatment. (**B**) The SPAD value of wheat regeneration leaf under MeJA, ETH, and ABA treatment. (**C**) Starch content of TN under MeJA, ETH, and ABA treatment. (**D**) Reducing content of TN under MeJA, ETH, and ABA treatment. The error bars indicate standard deviations.

The wheat samples were treated with ETH, JA, and ABA after mowing and the regrowth rate was measured. The results showed that the shoot regrowth rate increased after treatment with ETH and MeJA compared to the control (treatment with H_2_O) (Figure 13A). However, when plants were treated with ABA, regrowth was inhibited (Figure 13A). Although the regrowth rate increased in the ETH and MEJA treatments, the SPAD value of the new leaves decreased (Figure 13B). The SPAD value decreased similarly to the decrease under ABA treatment (Figure 13B). The starch and reducing sugar contents were then determined, and the results showed that under hormone treatment the starch and reducing sugar contents reduced more rapidly than under normal conditions (Figure 13C,D).

After treatment with various hormones, the expression patterns of genes associated with IAA, CTK, and GA synthesis and transport were altered. Nearly all genes showed varying degrees of upregulation or downregulation in response to ETH, MeJA, or AA treatments (Figure S6, Table S2).

## 3. Discussion

Dual-purpose wheat is grown in many countries, but its growth pattern after mowing remains unclear [3,39]. Without a better understanding of the regrowth mechanism of wheat after mowing or grazing, it is not possible to manage this crop for improved summer yield. This is why the adoption of dual-purpose wheat has stagnated. In this study, the regrowth mechanism of wheat was analyzed using molecular, cytological, and physiological mechanisms that are beneficial for the development of dual-purpose wheat. When the regeneration process is initiated, the plant (cells) cease the normal growth state and redistribute hormones and nutrients [40]. Therefore, because the initial pattern of cellular growth is altered during plant regrowth, the capacity to switch to new tissue develops in accordance with the needs of the plant. This process is accompanied by physiological changes, alterations in gene expression, and cellular structure changes [40,41].

Variations in shoot and root biomass reflect the carbon distribution patterns in plants after mowing. In this study, agronomic trait analysis showed that mowing stopped root growth while accelerating shoot growth, indicating that nutrition in wheat was primarily redistributed for shoot regeneration in the early period after mowing. This finding indicates that shoot regrowth after mowing is promoted at the expense of underground growth [42]. However, some studies have indicated no significant changes in root biomass after mowing [43]. Additionally, there are some studies also proved root biomass increases after mowing, and as mowing intensity increases, root biomass accumulation also increases [44]. Our study revealed a marginal decline in root biomass, similar to that reported by Paez-Garcia [45].

Plant leaf development involves the production and differentiation of leaf primordia, which eventually develop into mature leaves [46]. In the present study, paraffin sections revealed that the rate of new leaf formation in wheat accelerated after mowing, indicating a faster rate of leaf primordia formation. Chromosomal heterochromatin was diminished, indicating that cell metabolism and activity might have increased after mowing. Given that the leaves are the primary organs of photosynthesis, it is expected that the rate of leaf growth would be proportionately higher to the growth of other plant parts after injury; as a result, both the leaf weight and new leaf area increase, reducing the extinction coefficient, increases the absorption of light, and promoting photosynthesis [47]. Our results also showed that the reduced growth rate of the roots was associated with an increase in the rate of growth of the leaves However, the cell volume of the new leaves did not change, possibly due to the low temperature [48]. The tillering stage is characterized by wheat development; because the primary roots can absorb more nutrients than the secondary roots, the damaging effects of mowing are greater in the secondary roots [49]. Sub-microscopic structural analysis revealed that wheat secondary roots were structurally chaotic, and some organelles had vanished in the later period after mowing.

Mowing was found to induce the genes in *PAL* metabolism pattern to upregulate and catalyze phenylalanine to produce cinnamic acid, which is then converted into lignin and phenol catalytic action with 4CL [50,51]. In the present study, PAL and 4CL activities increased after mowing, which is similar to the results reported by Guan [52]. PAL activity was reported to increase after wounding in broccoli [52]. In the current study, the synthesis rates of lignin and phenol also increased as a result of elevated PAL and 4CL activities, with the result that the wheat was protected from further damage. In addition, some studies have shown that the *PAL* gene was upregulated under cold conditions [53]. In the present study, *PAL* upregulation might also be related to low temperatures. In the sugar metabolism pathway, the genes were upregulated, indicating that the rate of sugar degradation increased in TN after mowing. The β-Bmybase and β-glucosidase activities and related genes expression pattern in the TN and SR also reflected the utilization rate of starch and reducing sugar increased. After mowing, wheat let regeneration takes precedence over root growth. It was previously reported that β-glucosidase-mediated hydrolysis of glucose increases after plant tissue wounding, and this process is important in cell wall loosening and elongation [54]. In this study, the genes of bglX/B were upregulated and the activity of β-glucosidase increased, suggesting that β-glucosidase is important for cell wall modifications and is beneficial to wheat shoot regeneration. As the concentration of starch and glucose decreased, β-Bmybase and β-glucosidase activities declined after 72 h, as opposed to 2 and 24 h. Plants accumulate more energy for regrowth during the early mowing period. However, owing to the loss of the main photosynthetic organs, the rate of photosynthesis was lower than the respiration rate, resulting in a decrease in the content of starch and soluble sugar in the TN. A previous study showed that grazing significantly reduced the accumulation of reduced sugar and total sugar in alfalfa [54].

The DEGs in the nitrogen metabolism pathway were upregulated after mowing, suggesting that additional nitrogen elements were required for wheat regrowth. It has been reported that nitrogen metabolism is accelerated under abiotic stress [55]. Two main enzymes are involved in plant nitrogen metabolism, namely nitrate reductase and glutamine synthase [56]. In this study, the level of activity of both enzymes increased in the TN and SR, which induced the roots to absorb nitrogen from the environment, promoting the regrowth of the leaves [57]. NR is also the main synthetase pathway of nitric oxide, an important substance in wound signal transduction [58]. The increase in NR activity after mowing may promote plant nitrogen fixation and transmission of plant injury signals. According to previous studies, mowing can improve NR activity as well in other plant [59]. Moreover, an increase in glutamine synthase activity also promotes wheat nitrogen accumulation in TN and compounds more glutamate. Glutamate also plays an important role in protein structure [60]. According to Toyota et al. (2018), glutamate plays an essential role in wound signal transduction [61].

Amino acid metabolism is essential for plant regrowth. In this study, the genes of amino acid biosynthesis were upregulated in TN during the regrowth process, such as ilvE, IVD, and fadA, suggesting that the rate of branched-chain amino acid degradation accelerated, and the precursor substances for glucosinolate biosynthesis increased [62]. Methionine is a sulfur-containing amino acid that serves as a precursor to ethylene [63,64], and its content might increase with the upregulation of DEGs in the E1.14.17.4 metabolism pathway. Later results confirmed that the ETH content increased. The TCA cycle is the final metabolic pathway and metabolic link for sugars, lipids, and amino acids [65]. The products of the TCA cycle after metabolism not only provide energy for plant growth but also carbon and nitrogen fragments for shoot regrowth. MDH and CS are rate-limiting enzymes of the TCA cycle. After mowing, plant respiration is enhanced, not only providing energy for plant regeneration, but also enhancing plant defense mechanisms [66]. The results of the present study showed that wheat MDH activity increased, whereas CS activity decreased. This finding indicated s that mowing impacts the TCA cycle, which is responsible for the accumulation of oxaloacetic acid in TN. Oxaloacetate can be used as a substrate for phenylalanine synthesis, which is an essential substrate for numerous amino acids and secondary metabolites. MDH activity increased, and CS activity decreased, indicating that production of secondary metabolites and amino acids is increased during regeneration.

Wheat regrowth after mowing involves both leaf repair and active growth. In this process, endogenous contents of IAA, CTKs, and GA changed in the TN and roots of the wheat, similar to previous studies [67,68]. During leaf development, IAA content reaches a maximum at the leaf primordia formation position, and the GA content also increases [69]. However, in the present study, with leaf regrowth accelerated, the IAA content decreased. The cZ and tZ contents increased during leaf development [46]. The increase in cytokinin/auxin levels after mowing was beneficial for leaf growth, which was similar to the results of the present study [67,70]. In the present study, the ratio of CTK to IAA increased after mowing, which is beneficial for leaf regrowth. The auxin response factor *ARF*, auxin synthesis genes *YUCC* and *GH3.1*, were upregulated during leaf regeneration, indicating that the synthesis rate of auxin increased. The expression of CTK metabolism-related genes, *CISZOG* and *IPT*, and cytokinin content increased, indicating that the CTK synthesis rate might be higher than the CTK consumed. The expression of the *GA20ox2* and *GA30ox2* genes was upregulated for the sequential oxidation of GA into biologically active gibberellin, which is important for the elongation and growth of cells [18]. In the gibberellin signaling pathway, the MYB transcription factor *GAMYB* gene expression was also upregulated, which also reflected an increase in the gibberellin synthesis rate after mowing. These three hormones were also compounded in the root, while their content decreased, which might account for the hormones being transported into the TN for shoot repair.

After plant injury, ETH, JA, and ABA contents rapidly increase and play essential roles in plant defense and regrowth [19,21,25]. In our analysis of the wheat, we found that mowing enhanced the contents of these three hormones, which contributed to the transmission of the plant’s injury signal and improved defense against pathogenic bacteria. The findings of a previous study indicated that the wound signal quickly triggered *ERF* gene expression and ETH was rapidly produced, serving as a proxy for wound signal-induced auxin biosynthesis [71]. In a previous study it was shown that the ETH synthesis-related gene, *ASC2*, was rapidly upregulated within 30 min after leaf wounding in *Arabidopsis* [26]. In the present study, the expression of *ERF3*, *ERF8,* and *ACS2* was upregulated in TN, which is consistent with the results of previous studies. JA has been shown to be involved in plant regeneration, and could be a long-distance injury signal that transmits the wound signal from the wound site to the meristem, ultimately promoting IAA synthesis [21]. JA activates *ERF115* expression and stimulates apical regeneration [72]. Many genes are upregulated during JA-mediated regeneration, including *OPR* and *LOX* [72]. Our results confirmed this conclusion. ABA plays an important role in abiotic stress responses by inducing ROS synthesis [58]. A previous study indicated that ABA stimulates wound suberization in kiwifruit [73].

In this study, we used JA, ETH, and ABA to treat wheat after mowing and found that ETH and JA stimulated wheat regeneration. Previous research has shown that JA-pretreated hypocotyl explants potentiate de novo shoot regeneration in *Arabidopsis* [74] and ETH-pretreated could improve the regeneration of cultured cells [75]. The treatment of wheat with ABA reduced its regeneration capacity, which likely inhibited the synthesis of cytokinins and auxins. The expression levels of the genes were subsequently validated under low-concentration hormone therapy, and the results showed that low concentrations of ETH and JA promoted the expression of GA, IAA, and CTK metabolism genes; conversely, ABA inhibited the expression of these genes.

## 4. Materials and Methods

### 4.1. Plant Materials

The wheat line “XiNong 136” was chosen for this study because of its large shoot biomass in winter and strong regeneration capacity after mowing [76]. The wheat seeds were sown in pots (20 cm bottom × 25 cm height × 35 cm top) containing 5 L of pindstrup substrate (The Kingdom of Denmark); five seeds were sown in each pot. Once the seeds germinated, they were covered with a thin layer (0.5 cm) of soil. Later, when the seedlings had 4–5 leaves, the pots were buried in the field, with the mouth of each pot flush with the ground. Half of the wheat samples were chosen randomly, and the shoots were cut with a sickle once they had 5–6 tillers, leaving approximately 2 cm of wheat stubble in the field. Approximately 60 wheat shoots were randomly selected after mowing and treated with different hormones: ETH (5 mg/L), JA (10 μM/L), and ABA (25 μM/L). The tillering nodes (TN) were collected at 0, 2, 24, and 72 h, following the procedure reported by Cui [68].

### 4.2. Shoot and Root Phenotype Investigation and Analysis

The agronomic traits of the shoots and roots were investigated to reveal the growth patterns of wheat after mowing. In this study, the agronomic traits of 15 plants subjected to (treatment group, T) were compared with the traits of 15 plants under normal growth conditions (control, CK) at each time point. The entire root was removed from the pot and washed with running water until the root was clean. The leaf length of regeneration was measured with a Vernier caliper, and the surface area and root number were measured using a root scanner (LA-S, Hangzhou, China). The root length was determined using ImageJ software (version:1.8.0) [77]. The wheat roots were then stained with methyl blue, and the number of root primordia in the 20 cm primary root was counted. In the last step, the dry weight of the shoots and roots was measured using a scale (XT-A, Hengshui, China) after oven drying. The root/shoot ratio was calculated by dividing root dry weight by shoot dry weight. In this study, agronomic traits were investigated at 0, 1, 2, 3, 6, 12, 18, and 24 days after mowing.

### 4.3. Cytological Analysis of Tillering Node and Root Tip

To analyze the internal growth pattern of the shoots and roots, ten primary root tips (PRT) and ten secondary root tips (SRT), as well as five main stems of wheat were collected at 0, 3, 6, 12, 18 and 24 d after mowing. The sample lengths of the root tips and main stems were set to 0.5 cm. The TN linked to the main stem was collected and a paraffin section of the TN was prepared. The process for paraffin section preparation was as follows. All samples were fixed in 70% FAA stationary liquid for more than 8 h. After immobilization, the samples were rinsed three times with water and stained with hematoxylin for three days. Afterwards, the samples were dehydrated with alcohol, made transparent with xylene, dipped in paraffin, and sliced using a slicer (JY-202A, Beijing, China). To obtain the cross-cut samples of TN, the wheat leaves were stripped and counted, leaving only SAM and TN for the paraffin sectioning. When cross-cutting the TN paraffin section, the cuts began at the SAM and continued until the SAM section disappeared. The following TN paraffin sections were used to analyze changes in TN. The section thickness was set to 15 μm. The cells were photographed using a stereomicroscope (SZX16, OLYMPUS, Tokyo, Japan).

### 4.4. Submicroscopic Structure of Wheat TN and SRT

For submicroscopic structural observations, five SRTs and five TNs (linked to the main stem) were collected 0, 3, 6 and 12, d after mowing. The site near the SAM was chosen for the TN samples to minimize submicroscopic structural errors, and the site 1 cm from the root tip was chosen as the SRT sample. The samples were fixed with 2.5% glutaraldehyde solution (PH = 7.3) for more than 5 h and then washed four times with 0.1 M phosphate buffer. Samples were then fixed with 1% osmic acid (0.1 M, PH = 7.2–7.4) for 3 h, washed with phosphate buffer for 15 min, and the process was repeated four times. Subsequently, the samples were dehydrated twice using a gradient of ethanol for 10 min and repeated once more. Dehydration was followed by permeation with a resin before embedding the samples. The samples were then solidified in a drying oven. The temperature of the drying oven was set to 50 °C, and the solidification time was more than 48 h. After solidification, samples were sliced using an ultramicrotome (EMUC7, Weztlar, Germany) and stained with 2% uranium dioxide acetate for 15 min and lead citrate for 5 min. A transmission electron microscope (TEM; HT7800, Tokyo, Japan) was used to examine the samples. This experiment was performed at the large-scale instrument sharing platform for life sciences at Northwest A&F University (Yangling, China).

### 4.5. RNA Extraction, mRNA Library Construction and Sequencing

TNs were collected at 0 (T0), 2 (T1), 24 (T2), and 72 h (T3) after mowing, with 3 biological replicates for each treatment; each replicate contained 15 wheat and a total of 12 RNA sequencing samples. Total RNA was extracted and purified using the TRNzol Universal kit (TIANGEN, Beijing, China). A NanoDrop ND-1000 (Thermo Fisher Scientific, Waltham, MA, USA) and Bioanalyzer 2100 (Agilent Technologies, Santa Clara, CA, USA) were used to analyze the quantity, purity, and integrity of the RNA. To build the library, only RNA samples that met certain criteria (concentration > 50 ng/L, RNA integration number [RIN] > 7.0, 260/280 > 1.8, and total RNA > 1 g) were used. mRNA and non-coding RNAs (ncRNAs) were retained after rRNAs was removed using the Ribo-Zero rRNA Removal Kit (Illumina, San Diego, CA, USA). Short fragments of the enriched mRNAs and ncRNAs were created using fragmentation buffer and then reverse-transcribed into cDNA using random primers. cDNAs was used to create libraries. The protocols for library construction and sequencing followed the standard procedures provided with the NEBNextUltra RNA Library Prep Kit (Illumina). Sequencing was performed using an Illumina HiSeq 4000 at Gene De novo Biotechnology Company (Guangzhou, China).

### 4.6. Transcriptome Assembly and Annotation

Reads with adaptor contamination were removed using Cutadapt (https://cutadapt.readthedocs.io/en/stable/) (accessed on 25 February 2023) [78]. Screened reads were mapped to the wheat genome (Triticum aestivum, http://urgi.versailles.inra.fr/download/iwgsc/IWGSC_Ref-Seq_Assemblies/v1.0/) (accessed on 28 February 2023) using HISAT2 software (https://daehwankimlab.github.io/hisat2/, version: hisat2- 2.0.4) (accessed on 12 March 2023) and assembled with default parameters using StringTie (http://ccb.jhu.edu/software/stringtie/) (accessed on 15 March 2023) [79]. Gene Ontology (GO) and Kyoto Encyclopedia of Genes and Genomes (KEGG) pathway databases were used to annotate the genes. Raw transcriptomic data were uploaded to the National Genomics Data Center (https://ngdc.cncb.ac.cn/?lang=en) (accessed on 23 April 2023) and BioProject ID is PRJCA018312.

### 4.7. Analysis of DEGs

Gene expression levels were measured according to the number of kilobytes per million mapped reads (RPKM) in RNA-Seq analyses [80]. The DESeq2 (http://www.bioconductor.org/packages/releases/bioc/html/DESeq2.html) (accessed on 24 March 2023), DEGs were chosen with |log2 (fold change) | ≥ 1 and a *p*-value < 0.05 [81]. The DEGs were subjected to GO and Kyoto Encyclopedia of Genes and Genomes (KEGG) enrichment analyses. To further understand the function of DEGs, the KEGG pathway of significant enrichment was analyzed using the Omicshare website (https://www.omicshare.com) (accessed on 25 April 2023). The high enrichment of KEGG allowed the identification of the main biochemical, metabolic, and signal transduction pathways implicated in the DEGs.

### 4.8. RNA Isolates and Gene Expression Pattern Analysis

Real-time quantitative PCR (qPCR) was used to detect gene expression patterns. TNs and SRTs were collected at 0, 2, 24, and 72 h after mowing, according to physiological and biochemical determinations. The samples of TNs were collected at 2, 24, and 72 h after hormone treatment, five TNs were collected as one sample at each time point and this was repeated three times. RNA extraction and qRT-PCR were performed as previously described [82]. The wheat gene actin (Gene ID: AB181991) was used as a control. Data of the real-time qPCR were normalized with the 2^−∆∆Ct^ method [83]. Real-time qPCR primers were designed using OLIGO 7. The primers were then submitted to NCBI to check the specificity of the wheat.

### 4.9. Physiological and Biochemical Measures

Physiological changes in TN and SRT were analyzed in this study. Samples were collected at 0, 2, 24, and 72 h after mowing, and three wheat samples were collected, mixed as one sample, and repeated three times at each time point. The samples were washed with water until clean and then stored in liquid nitrogen. In this study, PAL and 4CL activities, amylase and glucosidase activities, nitrate reductase (NR) and glutamine synthetase (GS) activities, malate dehydrogenase (MDH) and citrate synthase (CS) activities, and starch and reducing sugar content were detected according to Li with some modifications [84]. Each physiological detection was performed in triplicate. The SPAD values of five regenerated leaves were detected using SPAD-502Plus (Konika-Minolta, Tokyo, Japan) after hormone treatment.

### 4.10. Wheat Endogenous Hormone Content Determination

Endogenous hormones are essential for plant growth. Hormone content was determined using HPLC-liquid chromatography (LC-30A, Tokyo, Japan) at the College of Horticulture of Northwest A&F University (Yangling, China). Approximately 0.5 g wheat tissue (TN and SRT) was collected and detected according to Almeida-Trapp with some modification [85]. In this study, seven hormones, cZ, tZ, IAA, ACC, ABA, GA3, and JA, were detected in the TN at 0, 2, 24, and 72 h after mowing.

## 5. Conclusions

Dual-purpose wheat has been used in many countries, but the wheat regeneration mechanism is still unclear. In this experiment, we revealed the regeneration pattern through the analysis of cell, transcriptome, and related genes expression patterns. The results indicate that mowing causes the wheat growth pattern changed with nutrients and energy being preferentially supplied to shoots for regeneration. Hormone content and related gene expression profiles were also altered during the wheat regeneration process.

## Data Availability

Not applicable.

## Supplementary Materials

The following supporting information can be downloaded at: https://www.mdpi.com/article/10.3390/ijms242015353/s1.
