# Supplementary figures and images for: Effect of Mowing on Wheat Growth at Seeding Stage
Source: Int J Mol Sci. 2023 Oct 19;24(20):15353. doi: 10.3390/ijms242015353 (PMC10607078; doi:10.3390/ijms242015353)

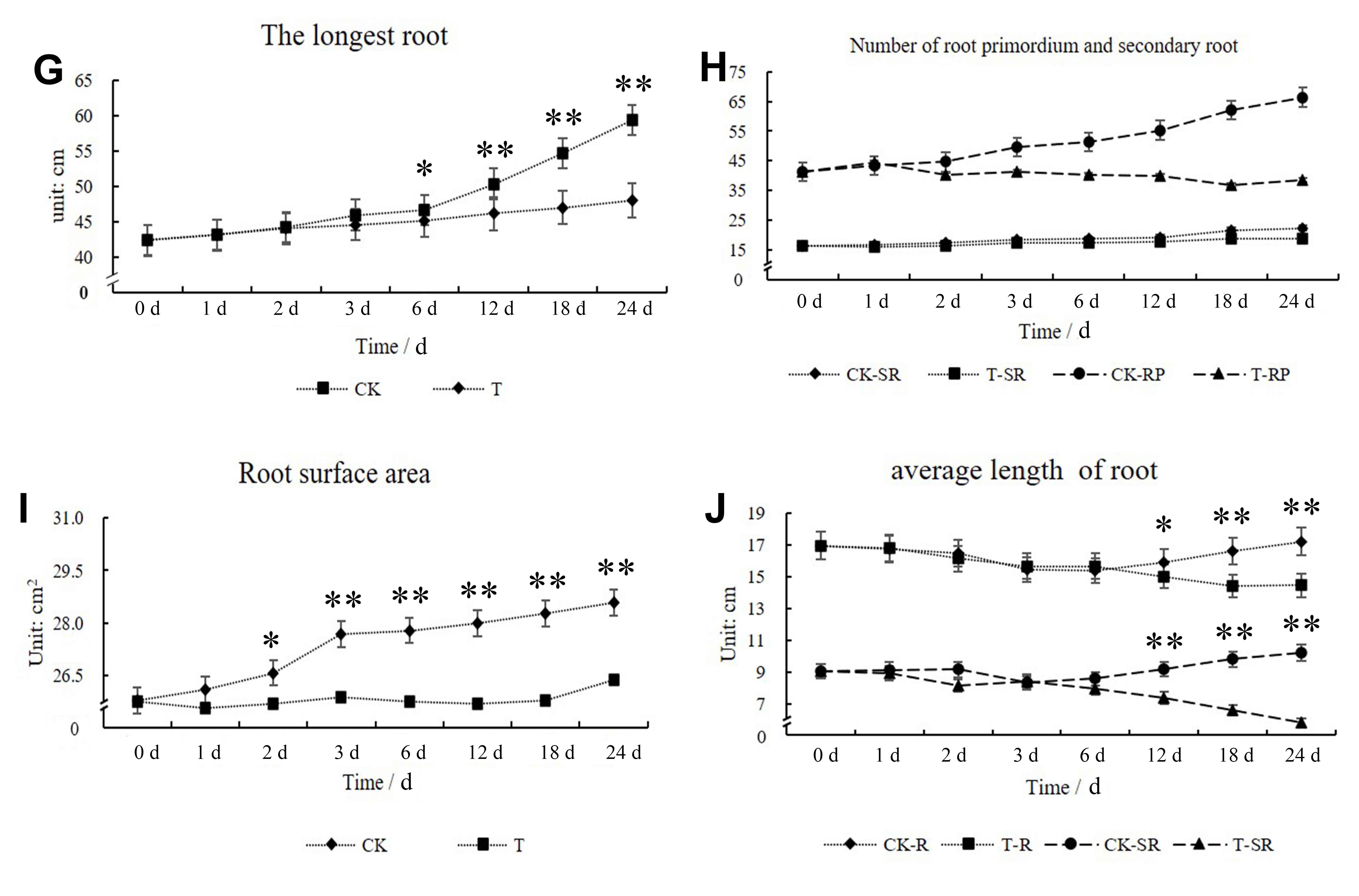

Supplement: Supplementary file 1 [file ijms-24-15353-s001.zip › Figure S1.jpg]

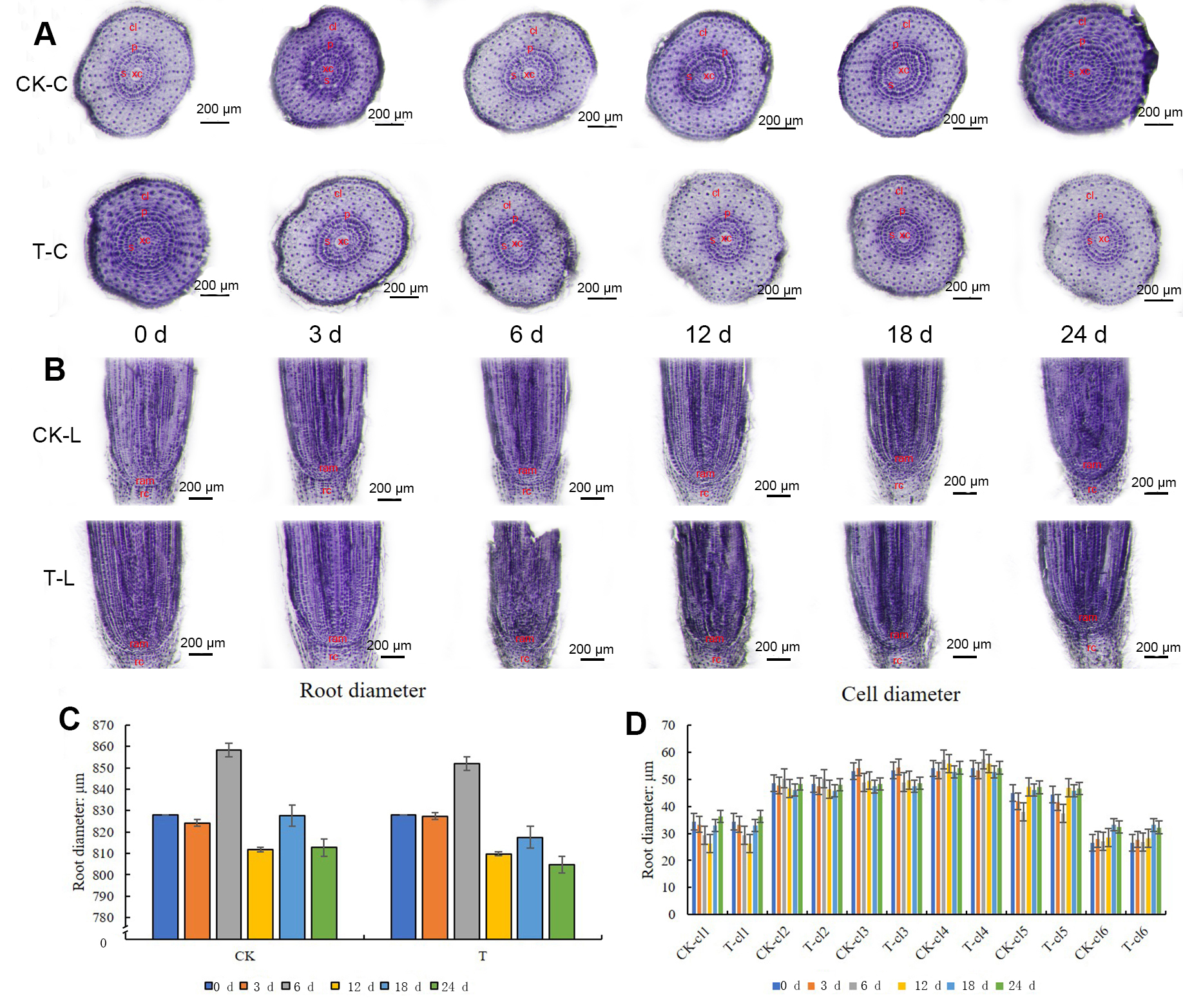

Supplement: Supplementary file 1 [file ijms-24-15353-s001.zip › Figure S2.jpg]

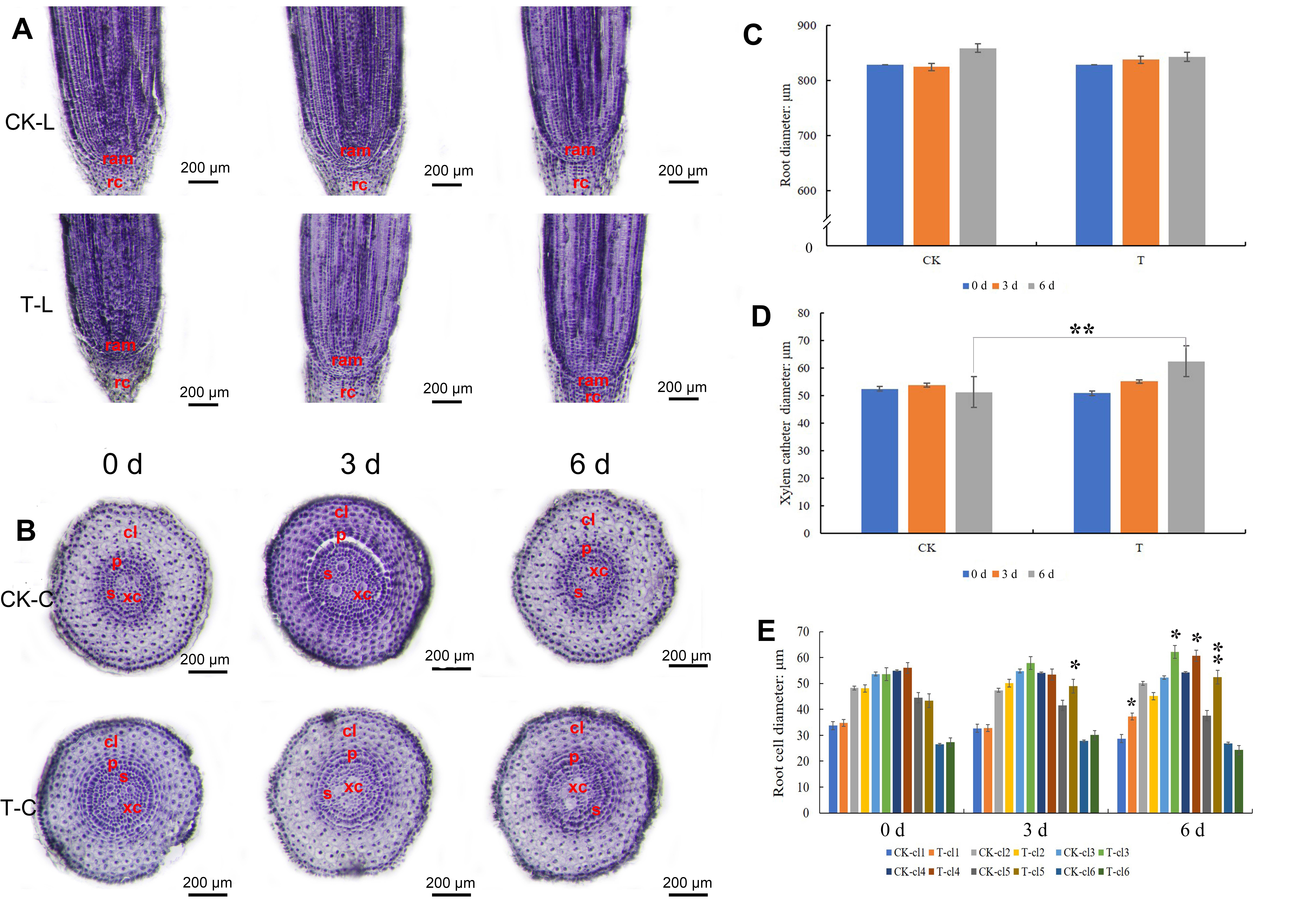

Supplement: Supplementary file 1 [file ijms-24-15353-s001.zip › Figure S3.jpg]

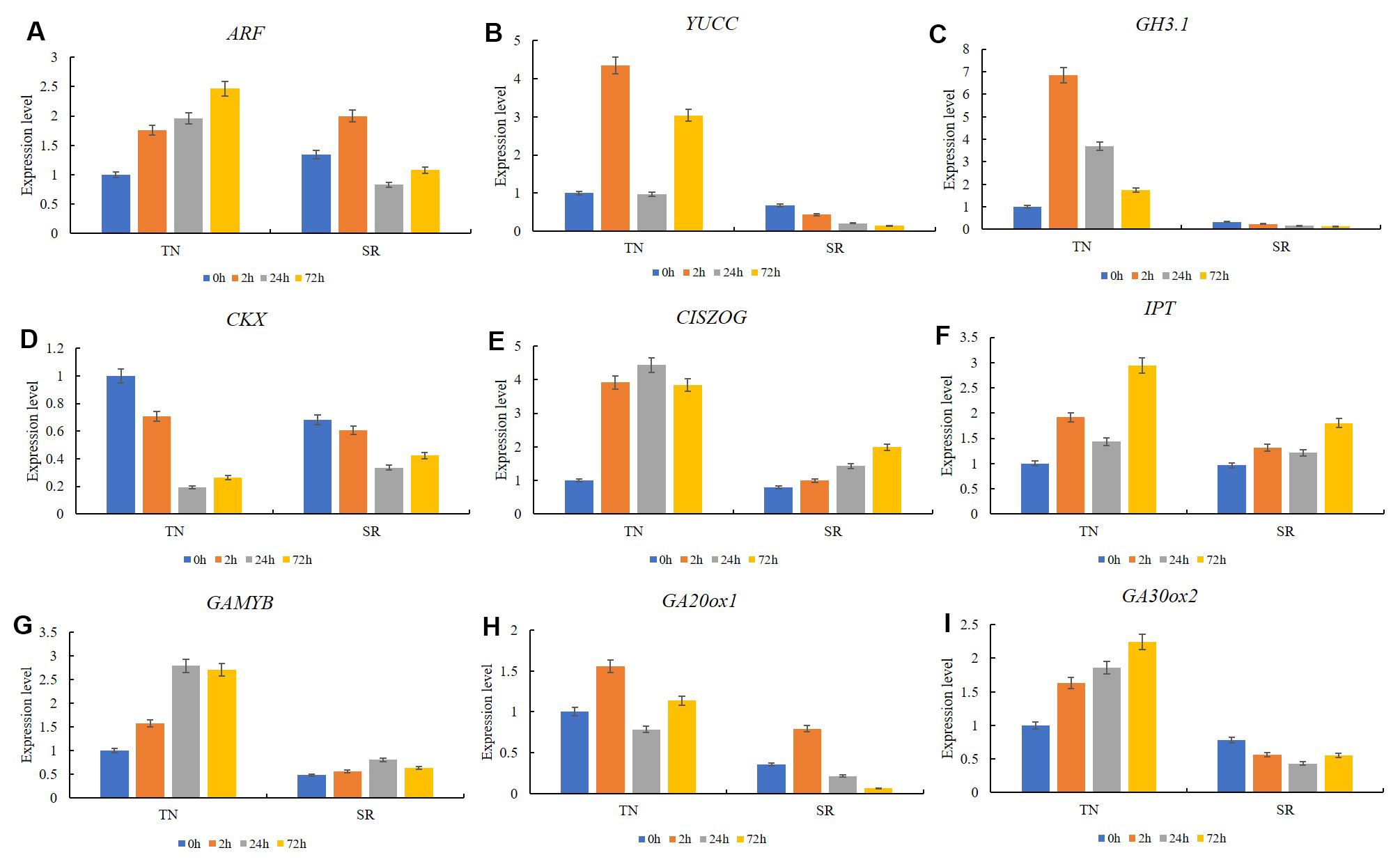

Supplement: Supplementary file 1 [file ijms-24-15353-s001.zip › Figure S4.jpg]

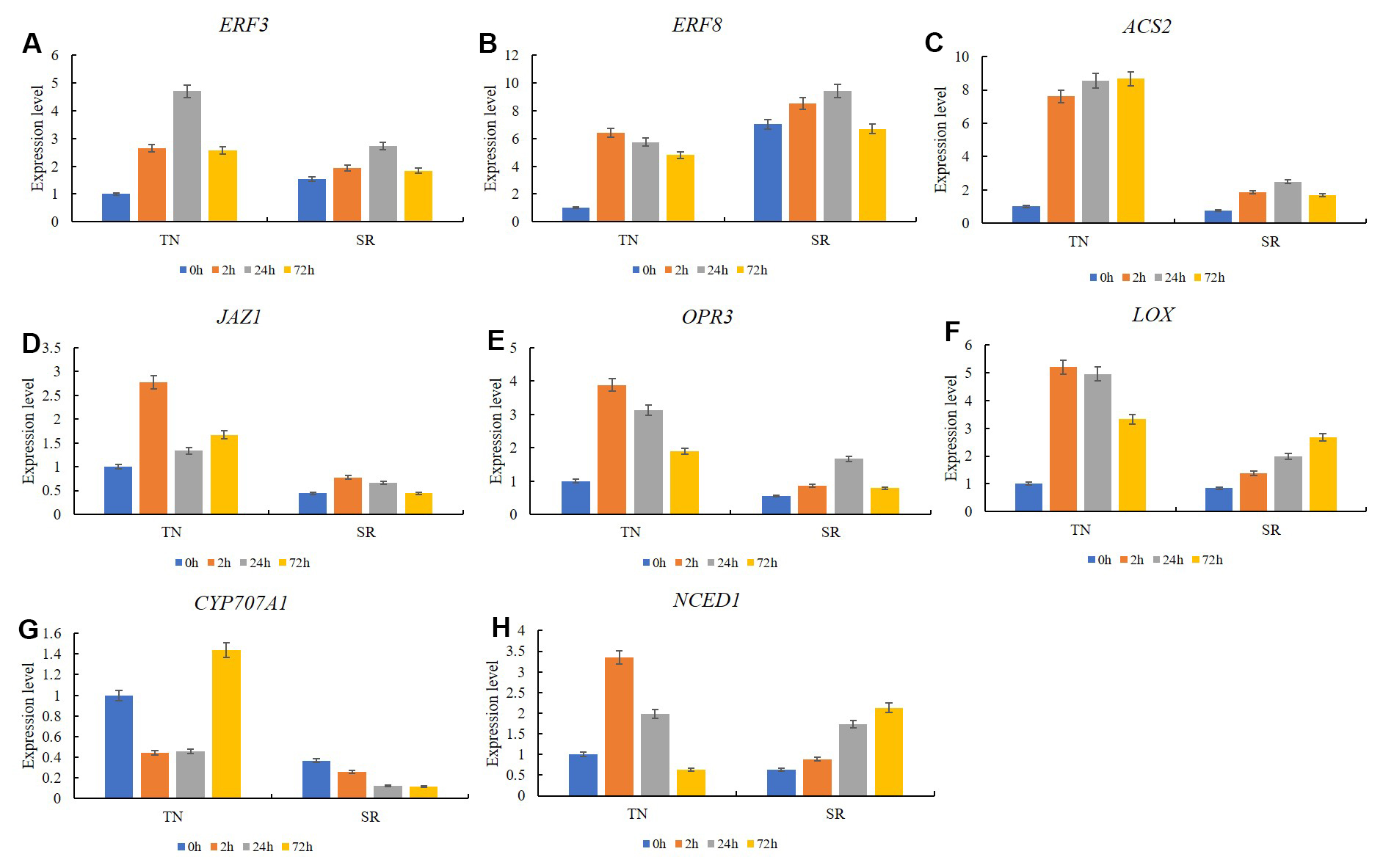

Supplement: Supplementary file 1 [file ijms-24-15353-s001.zip › Figure S5.jpg]

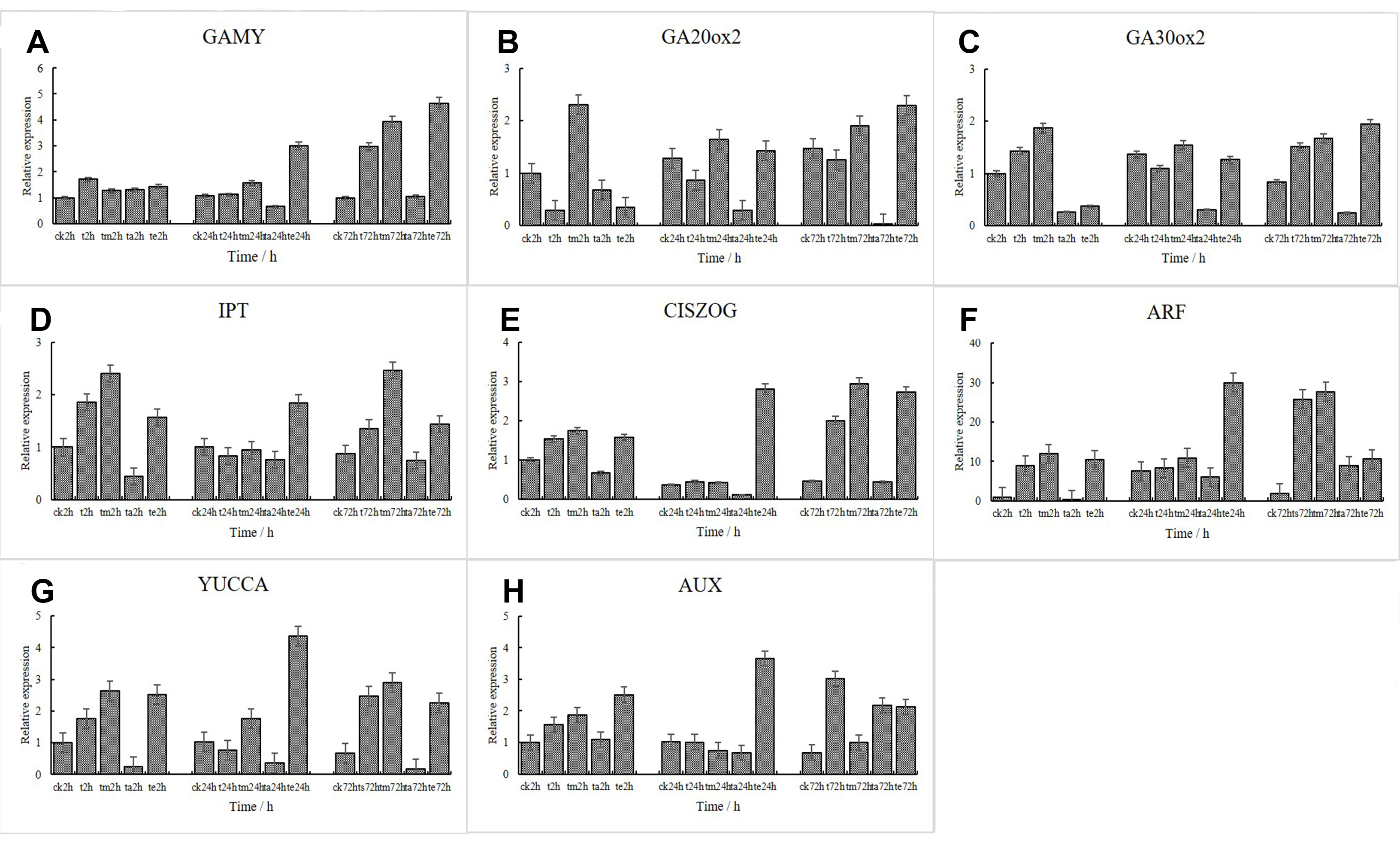

Supplement: Supplementary file 1 [file ijms-24-15353-s001.zip › Figure S6.jpg]
